# OB-fold Families of Genome Guardians: A Universal Theme Constructed From the Small β-barrel Building Block

**DOI:** 10.3389/fmolb.2022.784451

**Published:** 2022-02-11

**Authors:** Piero R. Bianco

**Affiliations:** Department of Pharmaceutical Sciences, College of Pharmacy, University of Nebraska Medical Center, Omaha, NE, United States

**Keywords:** OB-fold, SH3 domain, PXXP, genome guardian, SBB family, MCM, BRCA2

## Abstract

The maintenance of genome stability requires the coordinated actions of multiple proteins and protein complexes, that are collectively known as genome guardians. Within this broadly defined family is a subset of proteins that contain oligonucleotide/oligosaccharide-binding folds (OB-fold). While OB-folds are widely associated with binding to single-stranded DNA this view is no longer an accurate depiction of how these domains are utilized. Instead, the core of the OB-fold is modified and adapted to facilitate binding to a variety of DNA substrates (both single- and double-stranded), phospholipids, and proteins, as well as enabling catalytic function to a multi-subunit complex. The flexibility accompanied by distinctive oligomerization states and quaternary structures enables OB-fold genome guardians to maintain the integrity of the genome via a myriad of complex and dynamic, protein-protein; protein-DNA, and protein-lipid interactions in both prokaryotes and eukaryotes.

## Introduction

**FIGURE 1:**
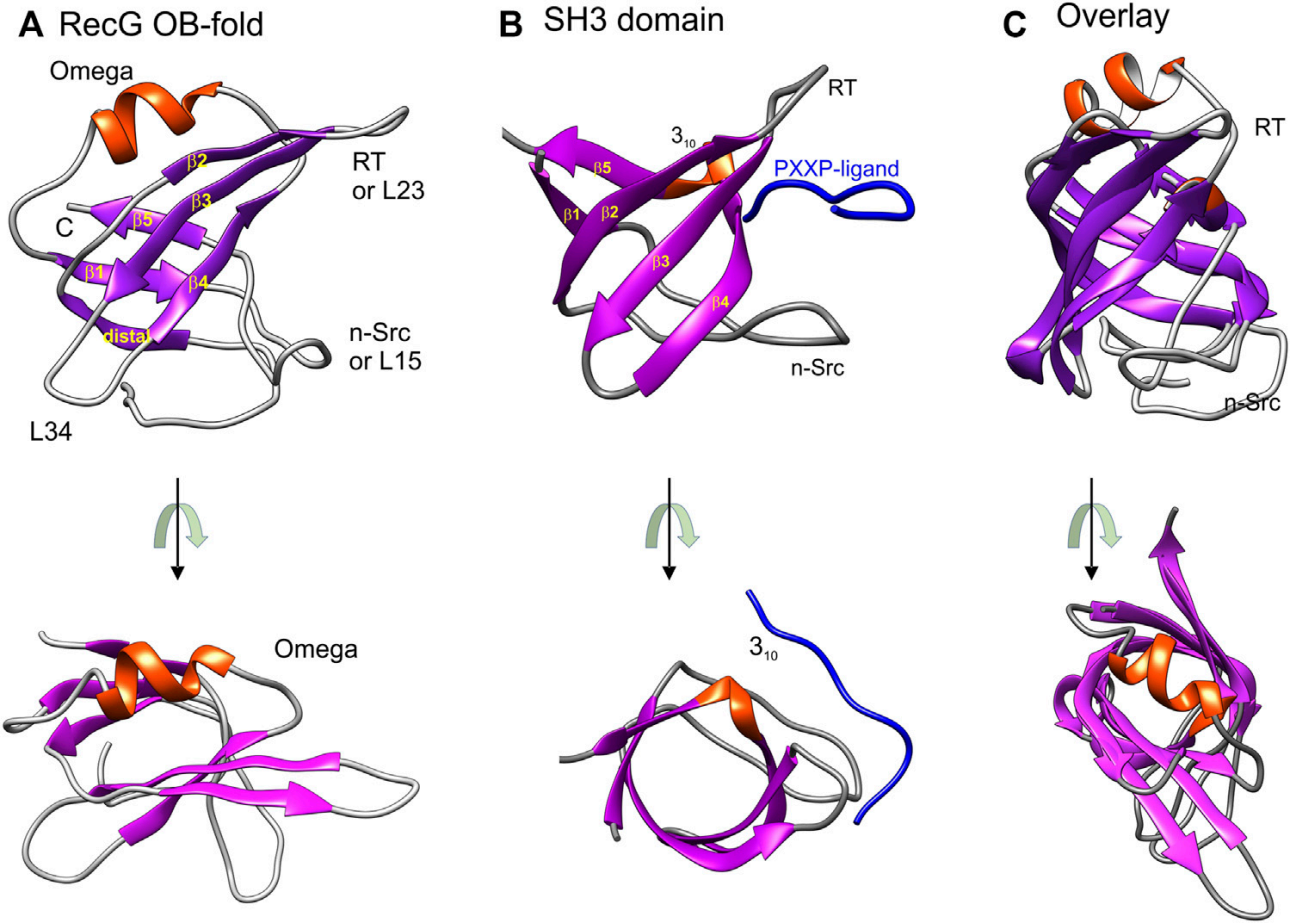
OB-folds and SH3 domains are structurally almost identical. Images were generated using Chimera with helices colored red and β-sheets in purple. **(A)** The OB-fold is from the *T. maritima* RecG (PDB file:1GM5) (Singleton et al., 2001). **(B)** The SH3 domain bound to a PXXP-ligand (blue) shown is from the ABL tyrosine kinase (PDB file: 1ABO) (Musacchio et al., 1994). The labeling of strands, helices, and loops in panels A and B is taken from reference (Agrawal and Kishan 2001). Loop nomenclature is from the Src protein (RT-Src and nSrc, respectively) (Yu et al., 1992). The RT-loop connects β2 and 3 (L23) while the nSrc loop connects β-strands 1 and 5 (L15). **(C)** Structural alignment of an SH3 domain (PDB file:2XKC) and with the RecG OB-fold. The alignment was done using TM-align (Zhang and Skolnick, 2005). This figure was adapted from (Bianco, 2021). In the images at the bottom of panels **(A–C)**, the representative OB-fold and SH3 domains are rotated towards the viewer so that the β-barrels can be viewed from the top down.

The small β-barrel (SBB) family of proteins is a large and ubiquitous family with diverse metabolic functions (Youkharibache et al., 2019). This family is comprised of members that contain a structurally conserved “urfold” consisting of five or six β-strands forming a domain that demonstrates flexibility in substrate binding ranging from phospholipids to proteins to RNA, single- and double-stranded DNA, as well as DNA of unusual structures, including the ssDNA regions G-quadruplexes and forked DNA molecules (Chen et al., 2018). This flexibility is provided by variations in the fold, unique modularity, as well as distinct oligomerization states and quaternary structures. The term “urfold” was proposed by Youkharibache *et al* to transcend and encompass superfold families including the closely related oligonucleotide/oligosaccharide-binding fold (OB-fold) proteins and Src homology 3 (SH3) domains (Figure 1). While these two superfamilies have different strand topologies, their structure is almost identical as when they are superimposed, they differ by less than 2 Å for the β-strands (Agrawal and Kishan, 2001; Bianco et al., 2017; Bianco, 2021). This structural similarity is critical to understanding OB-fold function and regulation as explained below.

**FIGURE 2:**
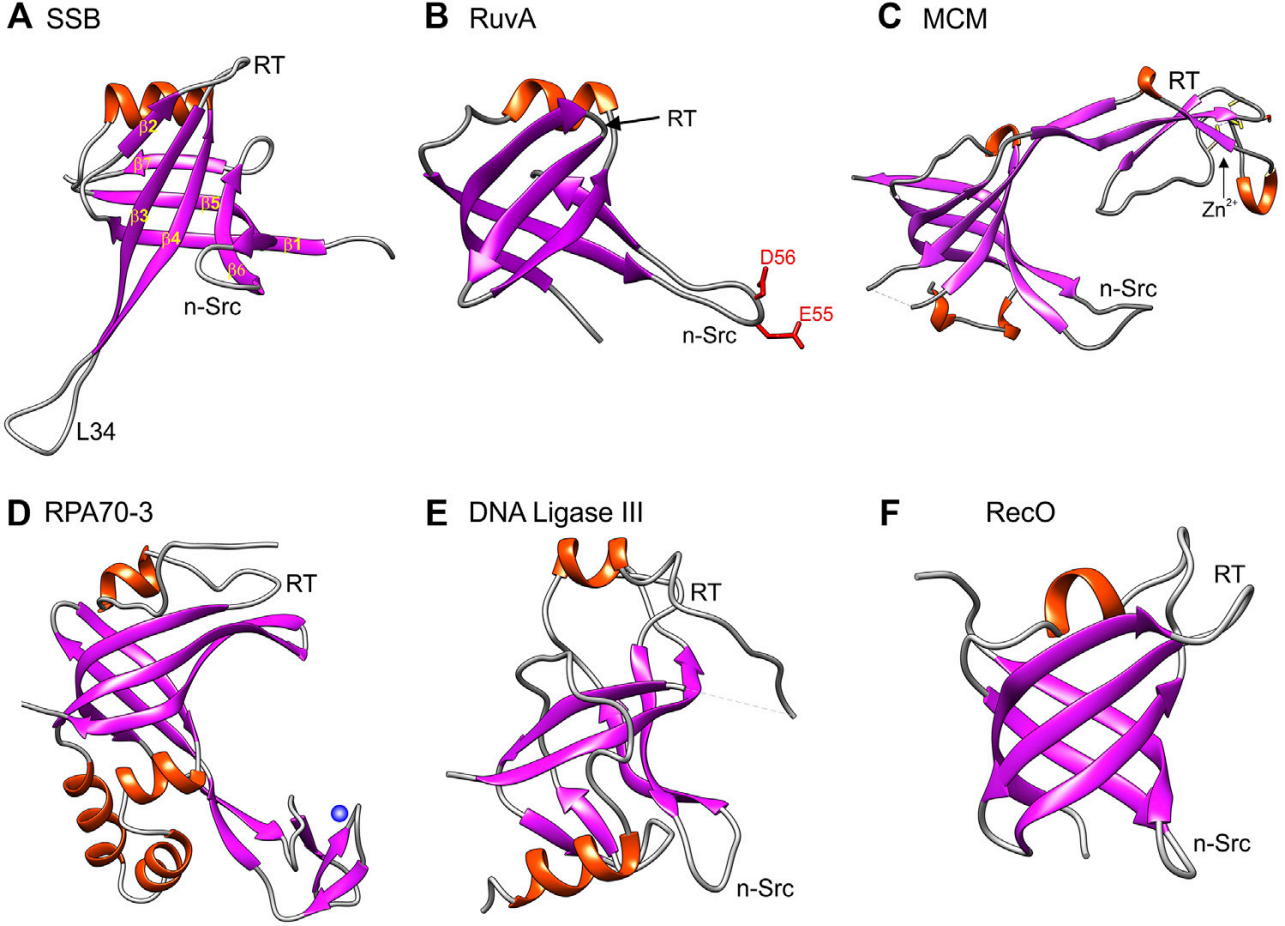
The OB-fold in genome guardians presents itself in different forms. Six representative OB-folds are shown. Panels A, B and F are prokaryotic while panels **(C–E)** are from eukaryotic proteins. As in Figure 1, images were generated using Chimera with helices colored red and β-sheets in purple. The orientation of each OB-fold is the same as in **(A)** such that the α-helix is located at the top of each structure to enable direct visual comparison. **(A)** SSB is the single-strand binding protein from *E. coli* (PDB file 1EYG); **(B)** RuvA is part of the *E. coli* Holliday Junction branch migration complex (PDB file 1C7Y); **(C)** The OB-fold from one of the subunits of a homohexameric MCM hexamer (PDB file 4ME3). **(D)** is the third OB-old present in the 70 kDa subunit of the eukaryotic Replication Protein A (functional homologue of *E. coli* SSB) (PDB file 4GOP); **(E)** is the OB-fold from DNA ligase III (PDB file 3L2P) and **(F)** is the OB-fold from RecO, a recombination mediator also from *E. coli* (PDB file 1U5k).

The OB-fold was originally identified as a novel folding motif in four unrelated proteins: a nuclease, a tRNA synthetase, and two toxins (Murzin, 1993). Since then it has been found in multiple proteins many of which are involved in genome stability (Amir et al., 2020; Bianco, 2021; Flynn and Zou, 2010; Nguyen et al., 2020). The OB-fold is comprised of two, three-stranded antiparallel β-sheets and is often described as a Greek key motif (Murzin, 1993; Singleton et al., 2001). The β-sheets are arranged to form a β-barrel that is typically capped by an α-helix at one end and a binding cleft at the other (Figure 1A). The loops that connect the β-strands vary in length, sequence, and conformation, contributing to the binding specificities of each OB-fold. Consequently, these domains also vary in size from 70 to 150 residues, and while the overall structure is conserved and structures align with an RMSD of 2.1 Å, conservation at the primary amino acid sequence level is notoriously low (Theobald et al., 2003). Finally, while the intrinsic structure of the OB-fold is maintained, its presentation and number of domains per protein or complex vary significantly and the substrate specificities for each domain are often different. This combined with the variations in loop sizes and composition, varying sequences, and the number of domains per protein or protein complex further contributes to the unique binding and enzymatic properties of each protein or protein complex. This is evident in the seven representative OB-folds (Figures 1A, 2).

For the *E. coli* single-strand DNA binding (SSB) protein, β-strands 3 and 4, as well as L34, are longer than those in RecG and the nSrc loop is twisted back towards the β-barrel (compare Figures 1A, 2A). As its name suggests, SSB binds to single-stranded DNA (ssDNA) whereas RecG binds to forked DNA substrates (Meyer et al., 1979; Molineux et al., 1975; Ruyechan and Wetmur 1976; Sigal et al., 1972; Slocum et al., 2007; Whitby et al., 1994). In contrast, RuvA binds to Holliday Junctions and in its OB-fold, the nSrc loop is longer and contains charged residues that are used for strand separation during branch migration (Ariyoshi et al., 2000; Iwasaki et al., 1992). Thus the RuvA OB-folds provide one catalytic function to the RuvAB branch migration complex. In contrast to RuvA, in the OB-fold of the eukaryotic minichromosome maintenance protein (MCM) subunit from *Thermoplasma acidophilum* (tapMCM), the RT-loop is extended and is interrupted by 3_10_-helix that is itself interrupted by a zinc-binding motif or Zn-finger (Figure 2C) (Fu et al., 2014). This insertion seen in MCM subunits was selected here for comparison to Replication Protein A (the eukaryotic equivalent of *E. coli* SSB) which binds to ssDNA and, DNA ligase III (Bochkarev and Bochkareva 2004; Iftode et al., 1999). In RPA and the ligase, their OB-folds are also interrupted by insertions and DNA ligase III uses its OB-fold to bind dsDNA (Bochkareva et al., 2001; Fan and Pavletich 2012; Yates et al., 2018). For the RPA70 OB-fold (also knowns as DBD-C), one insertion is a 28-residue, Zn-stabilized, three, β-strand structural domain that is involved in ssDNA binding (Fan and Pavletich 2012). In contrast, for the recombination mediator RecO, the β-barrel is compact, the RT-loop is comparable in size to that of RecG, and the nSrc loop is shorter (Figure 2F) (Ryzhikov et al., 2011). This small subset of OB-folds shows how the variations on the SBB theme enable a single fold to impart distinct DNA binding properties to unrelated proteins with unique functions. However, as alluded to above, OB-folds do not only bind DNA but also proteins and phospholipids enabling additional levels of control critical to genome stability (Zhao et al., 2019; Ding et al., 2020).

Early insight into how proteins could bind to OB-folds to compete with ssDNA binding came from the work of Agrawal and Kishnan who compared the structures of SH3 domains to OB-folds (Agrawal and Kishan, 2001). SH3 domains are ∼50 residue modules that are ubiquitous in biological systems and which often occur in signaling and cytoskeletal proteins in eukaryotes (Dalgarno et al., 1997; Kay et al., 2000; Ponting et al., 1999; Sudol 1998). The SH3 domain has a characteristic fold which consists of five or six beta-strands arranged as two tightly packed anti-parallel beta-sheets arranged into a barrel form and is almost identical in structure to the OB-fold (Figures 1B,C) (Agrawal and Kishan, 2001). Critical to their function (and germane to this review), SH3 domains bind PXXP-containing ligands in a pocket sandwiched between the RT-Src (RT) and nSrc loops (Figure 1B) (Yu et al., 1992; Musacchio et al., 1994). This pocket corresponds to the canonical ssDNA binding pocket of many OB-folds and this model of binding is frequently used to regulate and stabilize OB-fold partner proteins.

In this review, examples of OB-fold genome guardians are presented and how they bind to and modify different DNA substrates will be discussed. This is followed by sections on protein binding, and how this is used to stabilize genome guardians as well as enable their regulation. Finally, using the *E. coli* SSB interactome as an example, the control of OB-fold function in maintaining genome integrity will be presented. Here the competition between ssDNA and protein binding to control interactome partners will be illuminated. These discussions will highlight the unique aspects of each OB-fold and how the variability in this small domain is utilized to create families of proteins whose overall function is to guard the genomes of the organisms in which they are active.

### OB-folds in Genome Guardians

**FIGURE 3:**
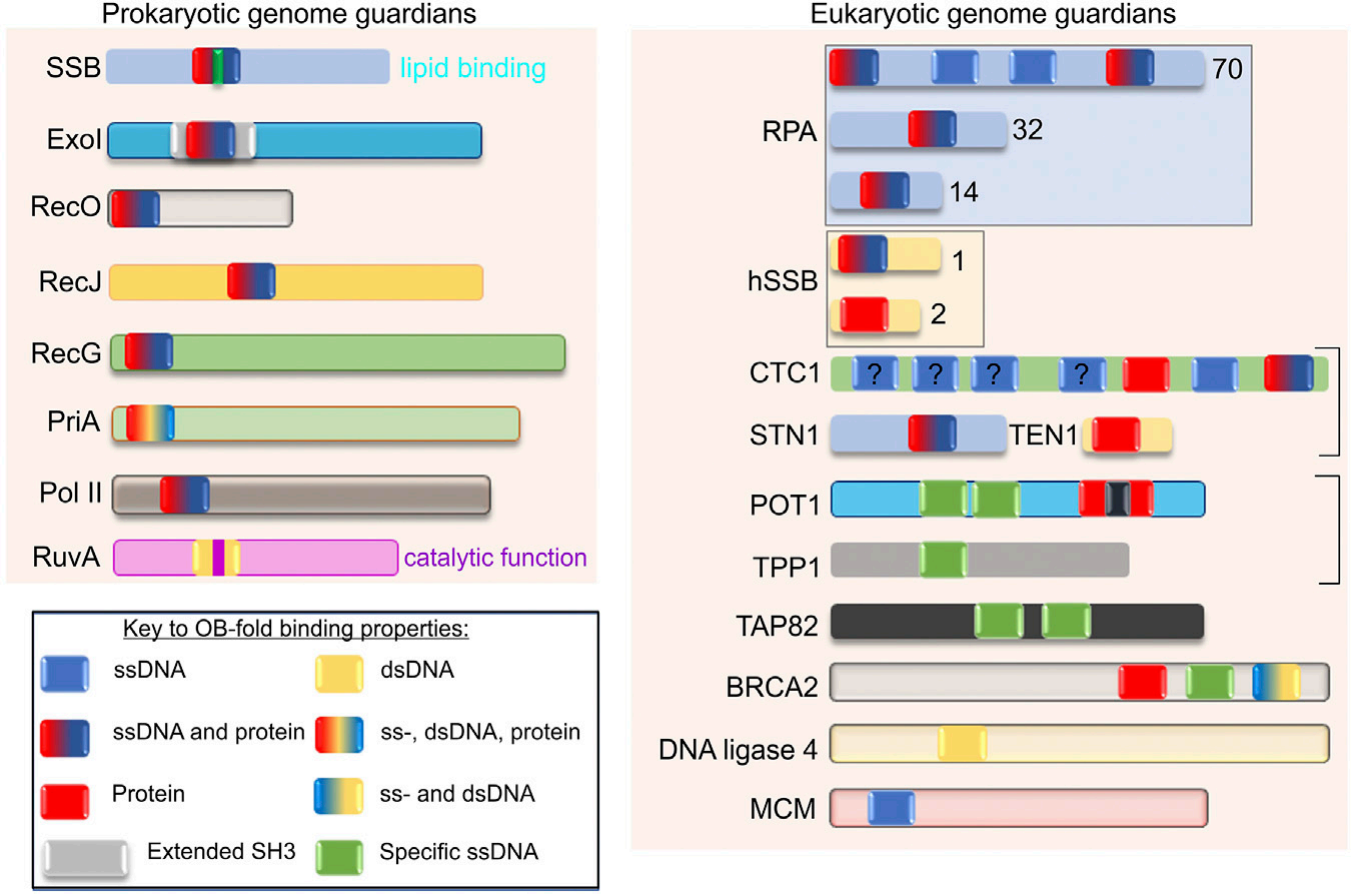
OB-fold genome guardians are ubiquitous. Schematics of representative family members from both prokaryotes and eukaryotes are shown. The figure is adapted from reference (Flynn and Zou, 2010). Each OB-fold is drawn the same size for simplicity and the different colours within each domain reflect the binding or substrate preferences ascribed to each protein. For Exonuclease I, the OB-fold is embedded within an extended SH3 domain (Breyer and Matthews, 2000). Family members are presented in different colors for clarity. The OB-folds of SSB, RuvA and POT1 have an additional coloured bar to indicate lipid binding, catalytic activity, and interruption by a Holliday Junction Resolvase domain, respectively. The “?” indicates an OB-fold of unknown function. The boxes indicate these OB-fold proteins exist in a complex. For details on the function of each protein the reader is referred to (Tye, 1999; Bianco, 2010; Flynn and Zou, 2010; Sarbajna and West, 2014; Wu et al., 2016; Nguyen et al., 2020; Bianco, 2021).

Proteins whose function is to maintain the integrity of the genome and safeguard it are classified as genome guardians. Many guardians such as the DNA helicase RecBCD and the recombinases RecA and Rad51 contain neither OB-folds nor SH3 domains (Chen et al., 2008; Conway et al., 2004; Singleton et al., 2004). However, the number of genome guardians utilizing OB-folds to mediate changes in DNA is increasing (Figure 3) (Agrawal and Kishan, 2003; Bochkarev and Bochkareva, 2004; Flynn and Zou, 2010; Gao et al., 2018; Nguyen et al. 2020). Recent work has shown that the SSB interactome is the first family of OB-fold genome guardians identified in *E. coli* (Bianco, 2021). However, SSB interactome members are not the only OB-fold proteins guarding the bacterial genome as shown for RuvA, which is not an interactome partner but contains OB-folds (Rafferty et al., 1996). In eukaryotes, the OB-fold family of genome guardians is large and likely to increase in size as additional structures are determined (Flynn and Zou, 2010). Thus the concept of OB-fold genome guardians is universal and at present includes at least 40 proteins and this list is likely to grow.

Representative members of the prokaryotic and eukaryotic OB-fold genome guardian families are shown in Figure 3. Included in this figure are the canonical single-strand DNA binding proteins, SSB in *E. coli,* and RPA and the human SSB1 complex from eukaryotic cells. There are also nucleases (Exo I and RecJ), recombination mediators (BRCA2 and RecO), DNA ligases, polymerases (Pol II), and helicases (PriA, RecG, and the MCM complex) as well as telomere end-binding (CTC1—part of the CST complex, POT1, TPP1, and TAP82) and branch migration complex proteins (RuvA). Visible inspection of the proteins selected, reveals that the number of OB-folds per polypeptide varies from one to as many as seven and the substrate-binding partner capabilities of each domain present per protein complex is also variable. This is perhaps best exemplified by CTC1 which has a total of 7 OB-folds (Lim et al., 2020). The first 4 have no demonstrated substrate specificity; the fifth or OB-fold E, binds protein exclusively and OB-folds 6 and 7 bind to telomere ssDNA and, ssDNA and protein, respectively. There are also examples of OB-folds such as those in SSB and RPA that bind to DNA non-specifically including tails of G4 quadruplexes, whereas POT1 and CTC1 proteins bind to sequence-specific ssDNA in telomere ends with high affinity (Wold and Kelly, 1988; Kim et al., 1992; Nandakumar et al., 2010; Ray et al., 2013; Rice et al., 2017; Shastrula et al., 2018; Lim et al., 2020). For the CST complex (which contains CST1) its DNA substrate-specificity is length-dependent: specific when ssDNA is short and non-specific as DNA length increases (Miyake et al., 2009; Hom and Wuttke, 2017). SSB, RPA70, POT1, and CTC1 contain OB-folds that bind to ssDNA but SSB and RPA bind to both ssDNA non-specifically and proteins, and, like SH3 domains, the SSB OB-folds also bind to acidic phospholipids (Fan and Pavletich, 2012; Zhao et al., 2019; Bianco, 2021). Within the domains that bind proteins, the mechanism of binding also differs, with some binding in the cleft formed between the RT and nSrc loops (POT1 and SSB) and others being partially wrapped by the binding partner (BRCA2) (Yang et al., 2002; Bianco et al., 2017; Chen et al., 2017; Rice et al., 2017; Ding et al., 2020). Finally, there are examples of proteins that bind to duplex DNA and again, their mechanism of binding is distinct. For RuvA, it binds to Holliday junctions while DNA ligases bind to nicked duplexes and MCM proteins bind to both ss- and dsDNA. This is explained in more detail in the next section (Iwasaki et al., 1992; Parsons et al., 1992; Ellenberger and Tomkinson, 2008; Tomkinson and Sallmyr, 2013; Shi et al., 2018).

The variability in OB-fold types is utilized by genome guardians to orchestrate the myriad of protein-DNA and protein-protein interactions required to maintain the integrity of the genome (Flynn and Zou, 2010; Amir et al., 2020). In the sections that follow, the mechanism of substrate binding by OB-folds and the ways that genome guardians use this binding to protect the genome are discussed. As there are so many genome guardians, it is not possible to discuss all possibilities. Instead, key proteins for which structures and biochemistry are available have been selected to highlight how the core of the OB-fold is used to guard the integrity of the genome (Arcus, 2002; Agrawal and Kishan, 2003; Theobald et al., 2003; Richard et al., 2009; Ashton et al., 2013; Wu et al., 2016; Nguyen et al., 2020; Bianco, 2021).

### OB-folds Interact With DNA Substrates in Unique Ways to Effect Distinct Outcomes

The variation in OB-folds suggests that proteins containing these domains may bind to DNA substrates in distinct ways to effect different reaction outcomes while maintaining genome integrity. To demonstrate how this can occur, 4 genome guardians were selected. The first is the *E. coli* SSB protein which is the canonical single-strand DNA binding protein (Meyer and Laine, 1990; Lohman and Ferrari, 1994). The second is DNA ligase III which binds to a nicked duplex and facilitates the sealing of the nick (Cotner-Gohara et al., 2010; Simsek and Jasin, 2011). RuvA, like SSB, is a tetramer but instead of having the OB-folds exposed to accommodate ssDNA, the folds are centrally located and interact with dsDNA during branch migration (Ariyoshi et al., 2000; Yamada et al., 2002). Fourth, the MCM DNA helicase forms a ring-shaped structure, and like RuvA, the OB-folds are positioned in the center of the ring and contact the ssDNA (Froelich et al., 2014).

*E.coli* SSB is the most well-studied single-strand DNA binding protein (Chase and Williams, 1986; Meyer and Laine, 1990; Kowalczykowski et al., 1994; Lohman and Ferrari, 1994; Shereda et al., 2008; Bianco, 2021). The role of this protein is to bind to exposed ssDNA and to as many as twenty partners that constitute the SSB interactome to regulate their activities concerning genome stability (Costes et al., 2010; Ryzhikov et al., 2011; Yu et al., 2016; Huang and Huang, 2018). The active form of SSB is a stable homo-tetramer (Sancar et al., 1981). Each monomer is divided into two domains defined by proteolytic cleavage: an N-terminal domain comprising the first 115 residues and a C-terminal tail spanning residues 116 to 177 (Curth et al., 1996). The tail is comprised of an intrinsically disordered linker and acidic tip (Lohman and Ferrari, 1994; Shereda et al., 2008; Kozlov et al., 2015; Bianco, 2017). For further details see the section “OB-fold regulation” including Figure 6.

**FIGURE 4:**
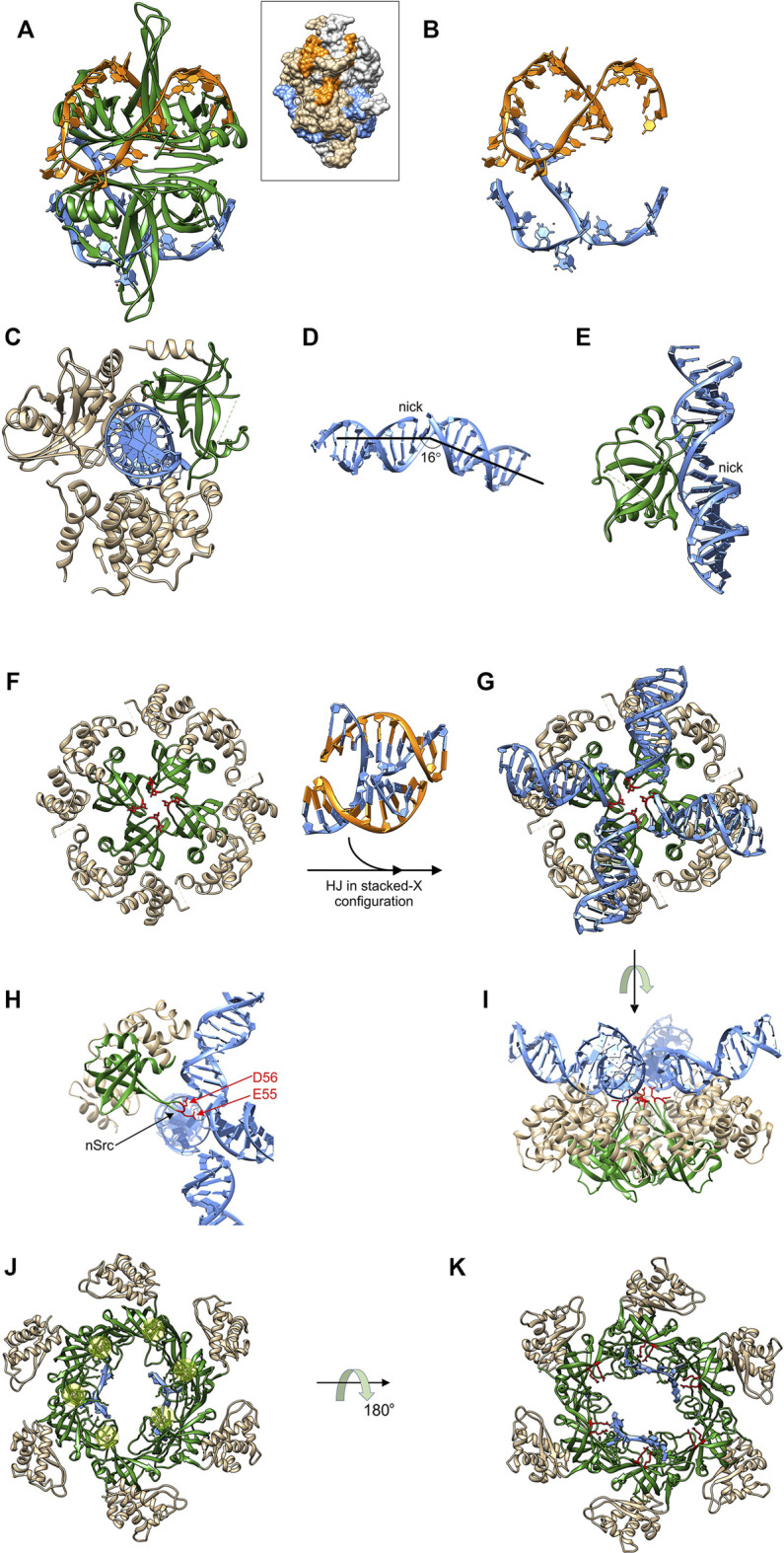
OB-folds interact with different DNA substrates and effect different outcomes. **(A)** the four OB-folds of SSB form an intimate complex with ssDNA (coloured orange and light blue) which is wrapped around the tetramer (PDB file: 1EYG). Inset: space-filling image to show how ssDNA is protected. DNA strand colouring is the same as in the ribbon diagram and SSB monomers are coloured light grey and neutral. **(B)** the ssDNA from the complex in **(A)**. **(C)** DNA ligase III utilizes its OB-fold (coloured green) to bind to the minor groove of dsDNA (light blue) opposite a nick [PDB file: 3L2P; (Cotner-Gohara et al., 2010)]. **(D)** The bent and underwound DNA from the structure in **(C)**. **(E)** A side view of the RT-loop of the DNA ligase III OB-fold interacting with the widened minor groove of the nicked duplex. **(F)**–**(I)**, The RuvA tetramer utilizes catalytic OB-folds to facilitate branch migration. Four images of the RuvA tetramer are shown [PDB File: 1C7Y; (Ariyoshi et al., 2000)]. The apo form is shown in **(F)** so that the four OB-folds (coloured in green) can be seen with the acidic residues indicated in red. **(G)** RuvA binds to a HJ (PDB File: 1M6G (Thorpe et al., 2003)), converting it into a planar X configuration. The orientation in G is the same as in **(F)**. **(H)** One subunit of RuvA is presented in a side view with the OB-fold in the same position as Figure 2B. Here, the extended nSrc loop places the acidic residues into the center of the junction. **(I)** These residues catalyze drive strand separation as arms of the Holiday Junction are translocated across the surface of the tetramer. Here translocation is driven by ATP hydrolysis in flanking RuvB hexamers (not shown). **(J)** and **(K)** The OB-folds in MCM proteins are arranged around the center of the ring to facilitate binding to ssDNA (PDB file: 4POG). In panel J, the protein-DNA complex is viewed from the top so that the positions of the Zn fingers can be seen (beneath the transparent yellow spheres). The positions of 2, ssDNA fragments are also visible in this view. In panel K, the complex is viewed from the bottom with highly conserved arginines coloured in red with those from 4 of 6 subunits in proximity to the ssDNA [R124 and 186; (Froelich et al., 2014)]. For further details of translocation in the CMG complex see (Meagher et al., 2019).

The N-terminal domains are visible in all crystal structures to date, are responsible for tetramer formation, and are almost exclusively OB-fold [Figures 2A, 4A; (Raghunathan et al., 2000; Savvides et al., 2004)]. ssDNA binding by this domain is non-specific and occurs *via* the wrapping of the polynucleotide around the SSB tetramer using an extensive network of electrostatic and base-stacking interactions with the phosphodiester backbone and nucleotide bases, respectively [Figure 4A and (Chrysogelos and Griffith, 1982; Kuznetsov et al., 2006; Raghunathan et al., 2000)]. Within this complex, ssDNA bound to the tetramer is wrapped and bound securely in the OB-folds where it is protected (Figures 4A,B). In addition to ssDNA binding, OB-folds are also responsible for binding to the linker region of nearby SSB tetramers resulting in cooperative ssDNA binding (Bianco, 2017; Ding et al., 2020). The linker, which has not been visualized in crystal structures to date, mediates protein-protein interactions using a mechanism similar to that employed by SH3 domains binding to PXXP ligands (Kaneko et al., 2008; Su et al., 2014; Huang and Huang, 2018; Nigam et al., 2018; Ding et al., 2020). Linker binding by the SSB OB-fold, its competition with ssDNA for binding, and the role this plays in protein function will be elaborated in the section “OB-fold regulation”.

DNA ligase III functions in nuclear and mitochondrial DNA replication and repair pathways (Sallmyr et al., 2020). Like other ATP-dependent eukaryotic DNA ligases and the widely used T4 enzyme, DNA ligase III contains a common catalytic region consisting of a nucleotidyltransferase domain and an OB-fold [Figure 4C; (Ellenberger and Tomkinson, 2008; Shi et al., 2018; Tomkinson and Sallmyr, 2013) (Cotner-Gohara et al., 2010)]. In addition, the enzyme also possesses an α-helical DNA binding domain that is critical to the DNA clamping mechanism (see below). In sharp contrast to SSB, these three domains encircle nicked, double-strand DNA with each making contacts with the duplex, thereby sequestering the 3′-OH and 5′-PO_4_ (Ellenberger and Tomkinson, 2008; Shi et al., 2018). This clamping or jackknife mechanism is conserved in other ligases and holds the dsDNA in a distorted conformation where the DNA is bent, underwound and the minor grove adjacent to the nick is significantly widened [Figure 4D; (Cotner-Gohara et al., 2010)]. The OB-fold via its RT loop binds to the minor groove opposite the nick, secures the DNA within the active site of the nucleotidyltransferase domain, and functions to position the nicked DNA substrate during all the remaining steps of the ligation reaction (Pascal et al., 2004; Cotner-Gohara et al., 2010). Thus for DNA ligase III, the role of the OB-fold is to participate in the jackknife mechanism and to bind to the minor groove of the duplex thereby positioning the DNA so that efficient ligation can occur.

The RuvA tetramer is intrinsic to the branch migration process catalyzed by RuvAB (West, 1997). In contrast to both SSB and DNA ligase III, it binds to intact DNA in the form of a Holliday junction (HJ). RuvA has several roles which include (i) changing the configuration of a Holliday junction to an open-square structure that is energetically more favorable for branch migration; (ii) targeting RuvB to the junction and stimulating its DNA helicase activity; (iii) coupling strand separation to duplex rewinding and (iv), facilitating binding of RuvC leading to resolution. Structural analysis of RuvA reveals that the protein consists of three domains. Domains I and II constitute the core of the protein and are responsible for tetramer formation and HJ binding [Figure 4F; (Ariyoshi et al., 2000; Nishino et al., 1998)]. Domain III is flexible, is not visible in the structures shown, interacts with RuvB, and modulates its ATPase and consequently its branch migration activity (Nishino et al., 1998; Nishino et al., 2000). Each RuvA monomer contains a single, N-terminal OB-fold in Domain I, with each contributing an acidic pin, comprised of residues E55 and D56, crucial to the branch migration process [Figures 4F–I; (Ingleston et al., 2000)].

HJs are dynamic structures that fluctuate between at least four different conformations in the presence of divalent metal cations, one of which is shown in Figure 4 between panels F and G (Hyeon et al., 2012; Joo et al., 2004; McKinney et al., 2003; Wyatt and West, 2014). RuvA binding halts these conformational dynamics converting the HJ into an open planar configuration a requirement for efficient branch migration [Figures 4G,I; (Gibbs and Dhakal, 2018; Lushnikov et al., 2003; Panyutin et al., 1995)]. In this configuration, the extended nSrc loop of each RuvA monomer is positioned in the center of the HJ in preparation for strand separation coupled to rewinding during the branch migration process (Figure 4H). Concurrently, the HJ is inclined 10° upwards from the ideal plane on the surface of RuvA (Figure 4I). Once two RuvB hexamers are bound to opposite ends of the RuvA tetramer, branch migration ensues and requires a screw motion and lateral pulling or pumping of the dsDNA, which passes through the center of the RuvB hexamers, and over the surface of the tetramer. Here RuvA uses the 4 acidic pins comprised of E55 and D56 contributed from the n-Src loop of each OB-fold to direct the path of each nascent single DNA strand through the complex (Stasiak et al., 1994; Rafferty et al., 1996; Ariyoshi et al., 2000; Ingleston et al., 2000; Putnam et al., 2001). Thus in this context, the RuvA OB-folds are providing an additional catalytic function to the RuvAB complex, that is strand separation and rewinding coupled to ATP hydrolysis-coupled dsDNA translocation by the RuvB hexamers.

The MCM proteins form a hexameric ring that in archaea is comprised of six identical subunits while in eukaryotes, the complex is a heterohexamer with subunits arranged in a specific order (Davey et al., 2003; Maiorano et al., 2006; Tye, 1999). The MCM complex assembles with five other subunits comprised of Cdc45 and GINS (Go, Ichi, Nii, andSan; five, one, two, and three in Japanese; consisting of Sld5, Psf1 (partner of Sldfive 1), Psf2 and Psf3), to form the replicative DNA helicase, Cdc45-MCM-GINS or CMG (Ilves et al., 2010). For all MCMs the OB-folds of each subunit are positioned within the center of the channel where they can interact with both ds- and nascent ssDNA (Figure 4J). Two parts of the OB-fold facilitate these interactions. The extended RT-loop of the OB-fold of MCM subunits is interrupted a 3_10_-helix which is itself interrupted by a Zn-finger (Figure 2C). A recent structure of the budding yeast *S. cerevisiae* CMG bound to a forked DNA revealed that the zinc fingers of each MCM, extend from the complex to contact the unwound duplex DNA ahead of the MCM ring (Yuan et al., 2020). The nascent unwound ssDNA interacts with the canonical OB-fold where highly conserved arginine residues extend from the barrel of the OB-fold and are thereby positioned in the center of the channel to make contact with the nascent ssDNA (Figure 4K). Thus in this case the OB-fold contacts both ss and dsDNA.

### OB-fold Regulation Is Central to Genome Stability

Modifications to the central β-barrel structure of the OB-fold allow proteins to bind to and modify DNA in a variety of ways that were unlikely to have been predicted when the structures of the first OB-folds were determined (Murzin, 1993). If left unregulated, DNA binding by these proteins could have disastrous consequences for genome stability as they could cause excessive strand separation and/or spurious melting of duplex DNA that otherwise might be lethal to the cell as suggested previously (Pant et al., 2004; Shokri et al., 2006; von Hippel and Delagoutte, 2001). It follows then, that binding must be regulated. This can be achieved in different ways with three examples of protein/OB-fold binding presented.

**FIGURE 5:**
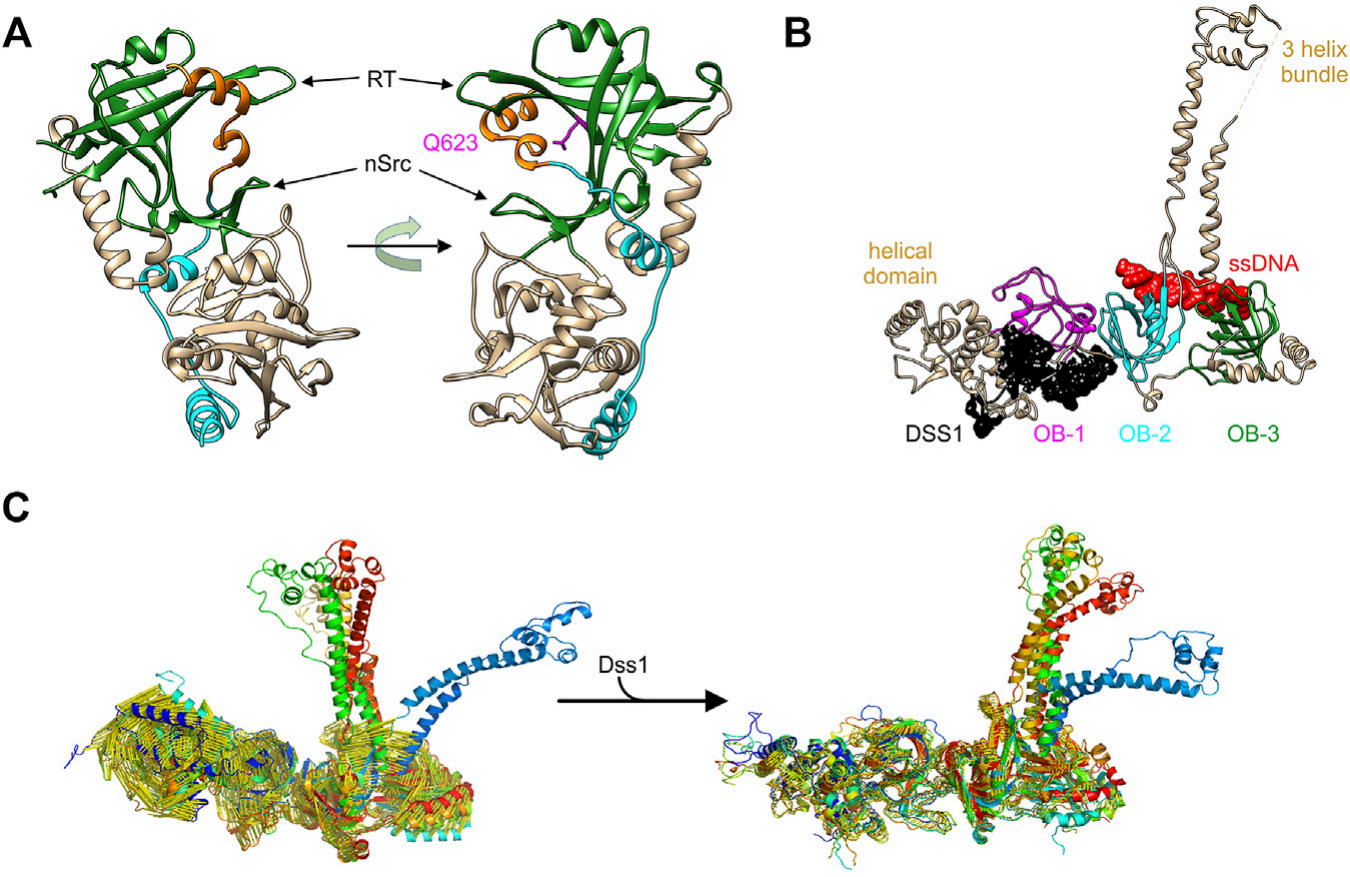
OB-folds bind to protein and DNA separately to control protein function. **(A)** The POT1-TPP1 complex (PDB file 5UN7). The third OB-fold of POT1 is coloured in green and the region of the TPP1-POT1 binding domain that sits in the canonical ssDNA-binding grooves is coloured orange. The orientation of the image on the left positions the OB-fold to a position similar to that of the OB-folds in Figure 3. The image on the right is rotated to show the location of the 3_10_-helix in its binding site and the position of Q623 (pink). **(B)** The structure of the three OB-folds and tower domain of BRCA2 are shown in complex with ssDNA (red) and DSS1 (black) (Yang et al., 2002) (PDB file 1MJE). **(C)** Molecular dynamics simulations of the apo-BRCA2 complex (left) and of the complex following DSS1 binding (right). These high-resolution images were provided by Dr. Bahadur, IIS, Kharagapur, India (Alagar and Bahadur 2020).

The shelterin complex is responsible for maintaining the integrity of telomeres (de Lange, 2005). In humans, this complex consists of six subunits, TRF1, TRF2, TIN2, RAP1, POT1, and TPP1 (Diotti and Loayza, 2011) Of these, POT1 and TPP1 contain OB-folds that are relevant to this section (Figure 3) (Liu et al., 2004; Theobald and Wuttke, 2004; Wang et al., 2007). POT1 and TPP1 function together by forming a stable heterodimer that protects chromosome ends and regulates telomerase activity (Wang et al., 2007; Xin et al., 2007). These two proteins bind one another via the protein binding domain of TPP1, also known as the POT1-binding motif (Figure 5A, left) (Chen et al., 2017; Rice et al., 2017). This interaction is crucial to POT1 function as it enables its localization to the telomere as well as regulating its binding. The structure of this complex reveals that, in addition to other interactions, the C-terminal one-third of the POT1 binding motif of TPP1 binds to the third OB-fold of POT1 (Figure 5A). A 3_10_-helix is located in the canonical, OB-fold ssDNA binding groove positioned between the RT and nSrc loops. The binding of TPP1 to POT1 stabilizes POT1 (Chen et al., 2017). This interaction is disrupted by mutations, with one of these, Q623, located within the POT1 OB-fold binding site for TPP1 (Figure 5A, right panel). When POT1-TPP1 binding is eliminated, POT1 becomes unstable with a shorter half-life, resulting in lower protein levels coupled to an activated DNA damage response at telomeres (Chen et al., 2017).

In mammalian cells, BRCA2 is a large and intricate example of OB-fold regulation within a single, multi-functional protein (et al. 2002; Thorslund and West, 2007; Thorslund et al., 2010; Shahid et al., 2014). BRCA2 binds to multiple protein partners and to DNA, to mediate the repair of DNA double-strand breaks and inter-strand cross-links by RAD51-mediated homologous recombination (Dray et al., 2006; Saeki et al., 2006; Le et al., 2021).

The structure of the C-terminal domain of the protein which is critical for the interaction with DNA revealed how binding and regulation could occur (Yang et al., 2002). This region of BRCA2 protein contains a helical domain and 3 OB-folds, with one interrupted by what has been called the tower domain (Figure 5B). The tower consists of two long, antiparallel helices capped by a three-helix bundle that has been proposed to bind dsDNA within the context of a tailed duplex (Schneider et al., 1998; Yang et al., 2002; Thorslund et al., 2010). The three OB-folds lie in close linear proximity, with two of them bound to ssDNA (red) and the third bound to the Deleted in split-hand/split-foot syndrome protein (DSS1; black). DSS1 is an intrinsically disordered, 70-residue peptide involved in multiple cellular functions including DNA repair (Marston et al., 1999; Kojic et al., 2003; Kragelund et al., 2016; Schenstrom et al., 2018). It is required for BRCA2 stability and the control of BRCA2 function in homologous recombinational repair (Li et al., 2006; Zhou et al., 2009). In the absence of DSS1, recombinational repair is virtually eliminated and this is due to increased degradation of BRCA2 (Li et al., 2006; Kristensen et al., 2010).

The binding of BRCA2 to an ssDNA/dsDNA junction is mediated by OB-folds 2 and 3 and likely the tower domain. The OB-folds bind to ssDNA while the tower is proposed to bind duplex DNA. This binding facilitates the nucleation of RAD51 filaments on the single-stranded tails of a processed, dsDNA break that are bound by RPA (Thorslund et al., 2010; Zhao et al., 2015). In addition to stabilizing BRCA2, DSS1 functions as an allosteric effector of BRCA2 and not as a DNA mimic as proposed (Alagar and Bahadur, 2020; Le et al., 2020; Zhao et al., 2015; Zhou et al., 2009). Here DSS1 binding to OB-fold 1 and the adjacent helical region results in structural changes in the C-terminal domain as well as the conversion of BRCA2 dimers into monomers (Alagar and Bahadur 2020; Le et al., 2020). It is conceivable that these effects are linked, but this has not been demonstrated. Using molecular dynamics simulations, Algar and Bahadur showed that the binding of DSS1 to the C-terminal tail of BRCA2 stabilizes this region (Figures 5B,C) (Alagar and Bahadur, 2020). This follows because apo BRCA2 (not bound to either DSS1 or DNA) showed a greater level of fluctuations in the helical domains and OB-folds 1 and 2, relative to the DSS1-BRCA2 complex. The effect of binding of DSS1 to OB-fold 1 may be propagated to OB-fold 2 and the tower, resulting in the restriction in conformational changes. In summary, the binding of an intrinsically disordered peptide to one OB-fold results in stabilization of protein structure and this influences both BRCA2 activity and possibly DNA binding as well.

**FIGURE 6:**
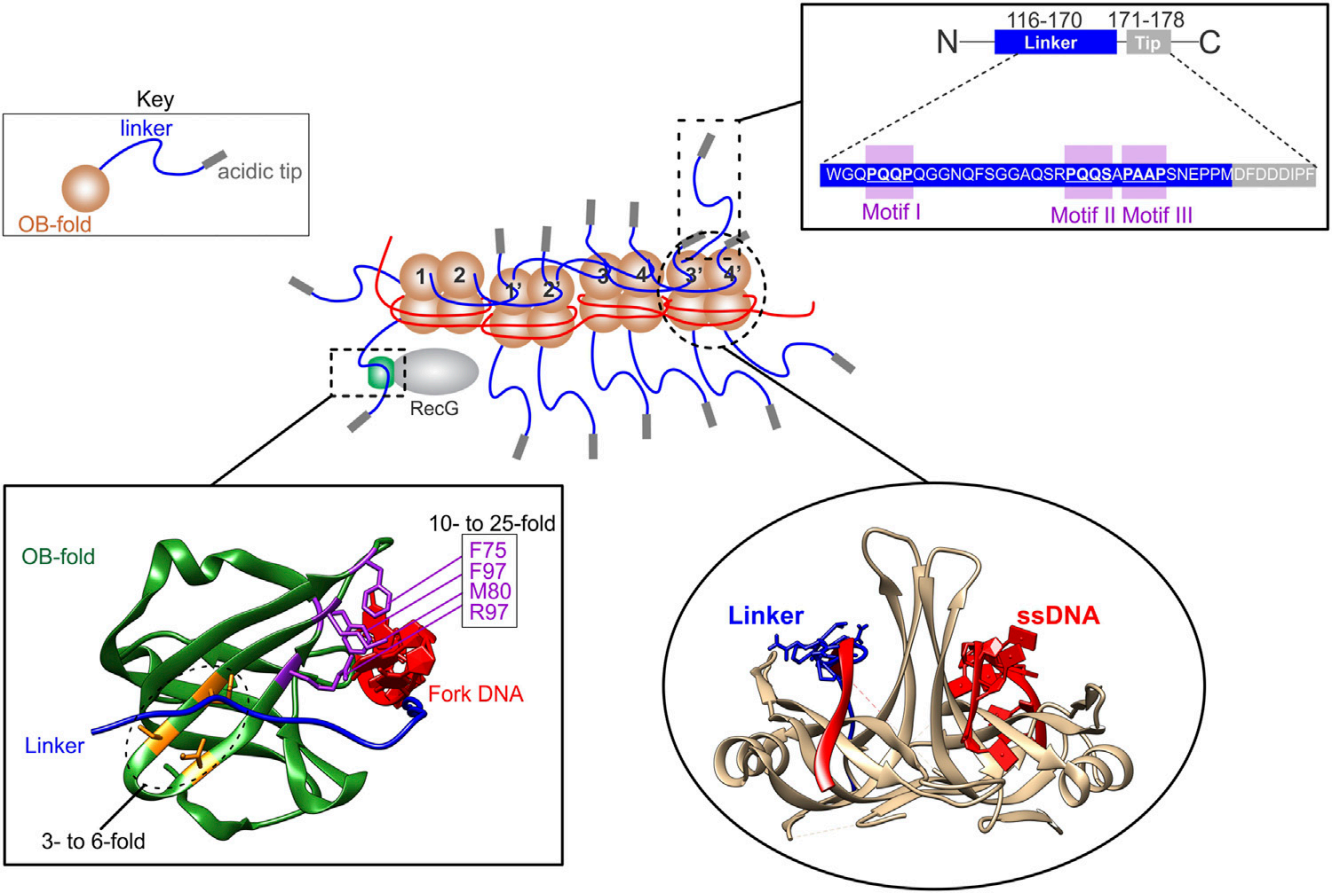
The SSB interactome is regulated by linker/OB-fold binding. A section of an SSB-ssDNA complex is shown in the center. Within this complex, the OB-folds (light brown) of some subunits are bound to ssDNA (red) while others bind to the linkers (blue) of adjacent SSB monomers. The regions of the linker responsible for mediating OB-fold binding are the PXXP motifs (inset, upper right). When bound to ssDNA, the linkers of monomers 1 and 2, bind to the OB-folds of monomers 1′ and 2′, respectively. Concurrently, the linkers of monomers 1′ and 2′ bind to the OB-folds of monomers 3 and 4, and their linkers bind to monomers 3′ and 4′, respectively. The ability of ssDNA and a peptide corresponding to the linker of SSB to bind to SSB OB-folds was initially shown using molecular modeling [lower right inset; (Bianco 2017)] and later by experiments [details in the text; (Ding et al., 2020)]. SSB linkers also bind to the OB-fold of partners as shown for RecG bound to the SSB-ssDNA complex with the structure of the RecG OB-fold in the lower left inset. Here the OB-fold is shown in green with relevant residues coloured in purple and orange. When mutated, residues in purple result in a 10- to 25-fold reduction in SSB binding *in vivo*, whereas those in orange result in only a 3- to 6-fold reduction (Ding et al., 2020).

The second example of an intrinsically disordered protein regulating OB-fold function is seen in the prokaryotic SSB interactome (Bianco, 2021; Lecointe et al., 2007; Shereda et al., 2008). Here, a 20-member, OB-fold, DNA-binding protein family is regulated by one member, the SSB protein whose OB-folds are in turn, controlled by acidic phospholipid, ssDNA, and protein binding in a competitive fashion (Ding et al., 2020; Harami et al., 2020; Zhao et al., 2019). The key region of SSB regulating interactome function is the intrinsically disordered linker or linker, which is positioned between the OB-fold and acidic tip of the protein (Figure 6, Key and inset top right). Here, the linker uses one or more of its conserved PXXP motifs to mediate protein-protein interactions by binding to the canonical ssDNA binding pocket positioned between the RT and nSrc loops of the OB-folds in either SSB or interactome partners where it competes with ssDNA (Figure 6, insets bottom right and bottom left). This binding forms the essence of the linker/OB-fold model while the tip functions as a regulator of the tail region and as a secondary protein binding site (Bianco, 2021; Ding et al., 2020; Zhao et al., 2019). The binding mechanism employed in the linker/OB-fold model to regulate the SSB interactome is similar to that used by SH3 domains to bind PXXP motifs to mediate target protein function (Bianco et al., 2017; Saksela and Permi, 2012; Yu et al., 1994). This follows because SH3 domains are structurally almost identical to OB-folds and, there are 3 PXXP motifs in the linker region of each SSB monomer (Figures 1C, 6, inset top right) (Agrawal and Kishan, 2001; Bianco, 2017).

The binding of SSB to ssDNA results in a conformational change in the protein so that the C-termini are more exposed (Kozlov et al., 2010; Williams et al., 1983). When ssDNA binding involves multiple tetramers, it occurs cooperatively and results in shortening of the DNA length (Chrysogelos and Griffith, 1982; Krauss et al., 1981; Ruyechan and Wetmur, 1975). The change in ssDNA length occurs because the polynucleotide is wrapped around each tetramer (Figure 4A). Concurrently, each tetramer also binds to its neighbors via linker/OB-fold interactions (Figure 6, center). Within this complex, some OB-folds bind to DNA while others bind exposed linker PXXP-motifs of adjacent tetramers [Figure 6, lower right; (Bianco, 2017; Ding et al., 2020)]. This results in an extensive network of linker/OB-fold interactions forming a stable complex that protects the ssDNA requiring elevated concentrations of salt or translocation by DNA motor proteins to disrupt them (Figure 6, center) (Lohman and Ferrari, 1994; Manosas et al., 2013; Green et al., 2016; Bianco, 2017; Bianco et al., 2017). Not surprisingly, mutation of the PXXP motifs eliminates cooperative binding to ssDNA (Ding et al., 2020).

The conformational change in the protein associated with binding of SSB to ssDNA also makes linkers available for interactome partner binding which facilitates these proteins being loaded onto the DNA, their functions regulated, and, in some cases, this is accompanied by SSB dissociation (Bell et al., 2015; Sun et al., 2015; Bianco et al., 2017; Nigam et al., 2018; Ding et al., 2020; Hwang et al., 2020; Wang et al., 2020). One example of an interactome partner is the RecG DNA helicase which binds to, and regresses stalled DNA replication forks into Holliday junctions (McGlynn et al., 2001; Singleton et al., 2001; Manosas et al., 2013; Lloyd and Rudolph, 2016; Bianco, 2020).

RecG has a single OB-fold in the wedge domain, responsible for fork binding (Mahdi et al., 1997; Singleton et al., 2001). This OB-fold binds to the linker of SSB, resulting in loading of the helicase onto DNA concomitant with the remodeling of RecG (Bianco et al., 2017; Ding et al., 2020; Sun et al., 2018; Sun et al., 2015; Tan et al., 2017). When the key residues of the linker/OB-fold interface, namely the PXXP motifs of SSB or separately, the OB-fold of RecG are mutated, SSB-RecG binding is eliminated (Ding et al., 2020). It is worth noting that when those residues that are part of the binding site of the helicase for the leading strand arm of the fork are mutated, SSB binding is reduced as much as 25-fold and fork binding is also eliminated (Figure 6, inset lower left) (Bianco and Lyubchenko, 2017; Briggs et al., 2005; Singleton et al., 2001). This is consistent with the model that that these binding sites overlap and that DNA and SSB binding is competitive (Bianco and Lyubchenko, 2017; Briggs et al., 2005; Sun et al., 2015). As the PriA and RecO OB-folds are essential for SSB binding, the linker/OB-fold model likely applies to all SSB interactome members which have an OB-fold as proposed (Figure 3; left side) (Kozlov et al., 2010; Inoue et al., 2011; Ryzhikov et al., 2011; Bianco et al., 2017; Ding et al., 2020; Hwang et al., 2020; Bianco, 2021).

### The Role of OB-folds in Fork Remodeling

The RecG and PriA DNA helicases are members of the SSB interactome, the first family of OB-fold genome guardians identified in prokaryotes (Bianco, 2021). Each of these proteins binds to SSB via linker/helicase OB-fold interactions, resulting in the loading of these enzymes onto stalled (RecG) or regressed (PriA) DNA replication forks, concomitant with their remodeling (Buss et al., 2008; Sun et al., 2015; Yu et al., 2016; Ding et al., 2020; Wang et al., 2020). These DNA helicases also use their OB-folds to bind to and alter or remodel the fork structure in unique ways.

**FIGURE 7:**
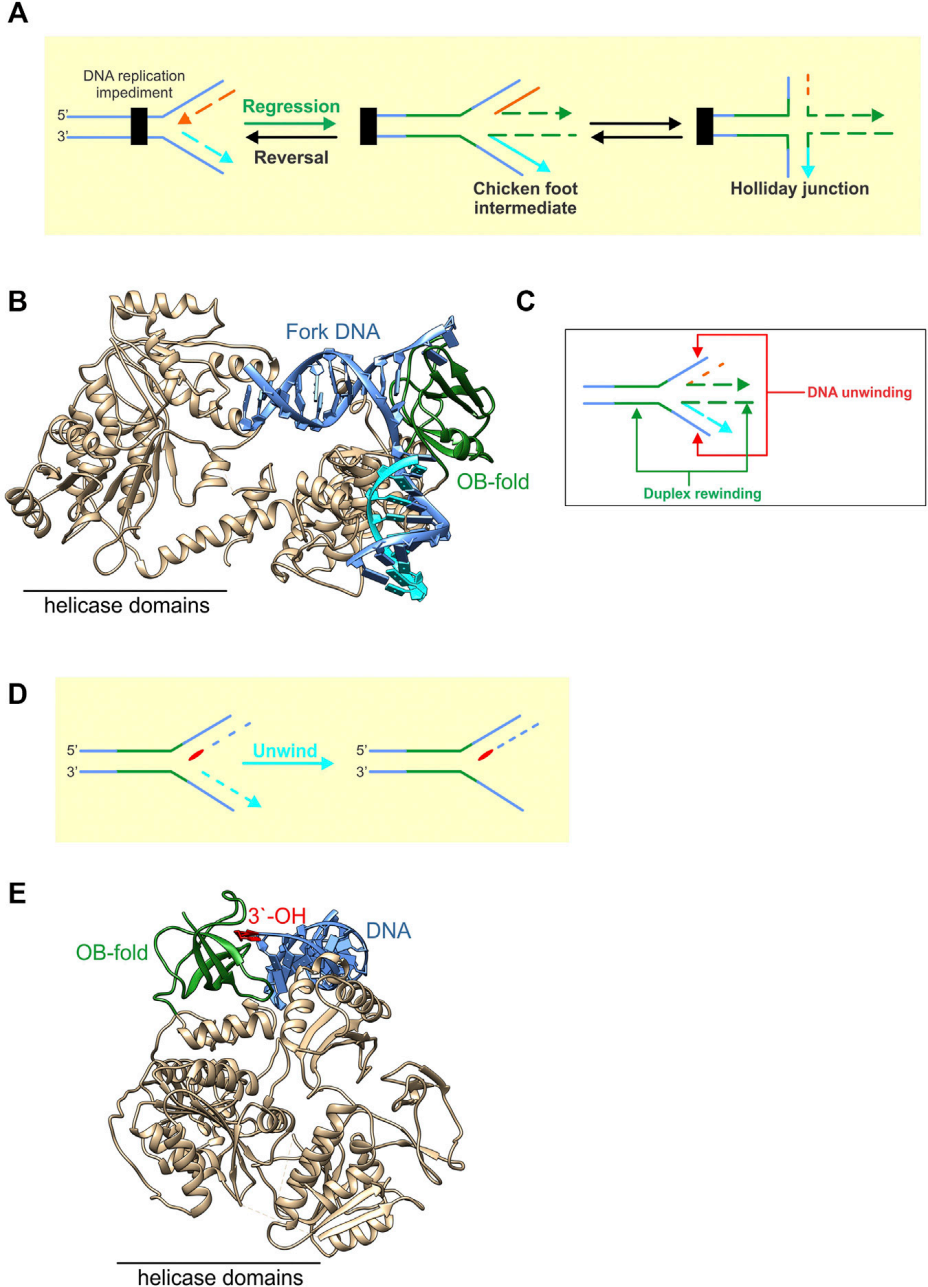
OB-folds in fork rescue helicases are used in different ways to modify fork structures. **(A)** RecG catalyzes fork regression, which is the net movement of the fork in a backward direction away from the site of a fork impediment. This results in the formation of a chicken foot intermediate or Holliday Junction. **(B)** RecG is shown as a ribbon diagram with the OB-fold coloured green and the remainder of the protein including the helicase domains, coloured neutral (PDB file 1GM5). The enzyme is bound to a fork with a gap in the nascent leading strand. ATP hydrolysis by the helicase domains is used to push the OB-fold through the fork. This results in the coupling of the unwinding of the nascent fork arms to the rewinding of DNA duplex both in the wake of advancing enzyme as well as ahead of the OB-fold a shown in the schematic in panel **(C)**. **(D)** PriA is shown bound to the leading strand arm of the fork (PDB file 6DGD). Here it utilizes its OB-fold (coloured green) to bind to the 3′-OH group (red) positioned at the fork with high affinity. This enables PriA to unwind the nascent lagging strand arm of the fork (light blue) so that the replicative helicase DnaB can be loaded onto the exposed ssDNA of the template lagging strand as shown in the schematic in panel E.

RecG catalyzes fork regression, where a stalled DNA replication fork is moved in a backward direction, away from the site of DNA damage, resulting in the formation of a Holliday Junction (Figure 7A). To do this, the helicase domains bind to the parental duplex DNA ahead of the fork while the OB-fold binds to the fork with high affinity (Figure 7B) (Singleton et al., 2001). The helicase domains use the energy from the hydrolysis of ATP and product release to generate >35pN of force to push the OB-fold through the DNA (Manosas et al., 2013). The OB-fold then couples DNA strand separation to duplex rewinding both in the wake of the advancing enzyme as well as ahead of it, resulting in Holliday junction formation (Figure 7C). This process occurs at an average rate of 269 ± 2bp/sec and processivity of 480 ± 20 bp (Manosas, Perumal, Bianco, Ritort, Benkovic and Croquette 2013). How the RecG-OBfold binds to forks is distinct from that of the MCM hexamer. For RecG, the barrel interacts with and splits the arms of the fork to facilitate strand separation followed by rewinding [Figure 7B; (Singleton et al., 2001)]. For MCMs, the extended RT-loop binds to duplex DNA while the OB-fold barrel binds to nascent ssDNA [Figures 2C, 4J,K; (Meagher et al., 2019)].

PriA binds to forks once RecG has catalyzed regression and/or additional processing has taken place to restore the fork structure (Marians 1999; Marians 2000). In contrast to RecG, PriA uses its OB-fold to bind to the 3′-OH group on the nascent leading strand arm of the restored fork with high affinity (Figure 7D, red base) (Mizukoshi et al., 2003; Sasaki et al., 2007) (Mizukoshi et al., 2003; Nurse et al., 1999). This binding is critical to both the activation of the ATPase activity as well as efficient ATP hydrolysis and is significantly enhanced by SSB (Manhart and McHenry, 2013; Tan and Bianco, 2021; Tanaka et al., 2007). This serves to enhance the ability of PriA to discriminate the correct fork structure by as much as 140-fold, orienting the DNA helicase on the fork so that it can unwind the nascent lagging strand arm (Figure 7E). Duplex unwinding ensures that the preprimosome (a complex of PriA, PriB, DnaT, PriC, DnaB, and DnaC) can be loaded onto the template lagging strand and that replication restart proceeds in the correct direction (Lee and Marians, 1987; Jones and Nakai, 1999; Jones and Nakai, 2001). This involves the loading by PriA, of the replicative DNA helicase, DnaB onto the lagging-strand template *via* a complex series of protein-protein interactions reminiscent of primosome (preprimosome + primase) assembly for ϕX174 DNA (Masai and Arai, 1996; Jones and Nakai, 1999; Marians, 1999; Marians, 2000).

## Summary

OB-fold genome guardians are essential proteins that perform a myriad of functions to maintain the integrity of the genome. To facilitate these functions, the small β-barrel (SBB) at the heart of the OB-fold structure is modified by the addition of loops and or additional domains to create domains with distinct properties. The domain is then placed in a unique position or positions in each protein so that diverse DNA substrates can be processed and/or protected in the correct way to facilitate genome stability. The family of OB-fold genome guardians is now known to extend to both eukaryotic and prokaryotic members, reinforcing the importance of these proteins in the maintenance of genome integrity in all organisms (Bianco, 2021).
